# The Hospital Nursing Supplement

**Published:** 1891-05-30

**Authors:** 


### "The Hospital" Nursing Mirror.

Being the Extra Nursing Supplement of "The Hospital" Newspaper.

Contributions for this Supplement should be addressed to the Editor, The Hospital, 140, Strand, London, W.C., and should have the word "Nursing" plainly written in left-hand top corner of the envelope.

### En Passant.

REGISTRATION OF MIDWIVES.—The *British Medical Journal*, which has been promoting the Midwives' Registration Bill against the general practitioners, announces that it is withdrawn "in view of the virtual pledge given by the Lord President of the Council to grant a select committee for the consideration of the whole subject next Session." In the same paper we learn that the Registration of Midwives has just become law in America; evidently the old country is getting behind the times.

NURSING AT THE HOMŒOPATHIC.—The 41st report of the Homœopathic Hospital, Great Ormond Street, states that a Special Committee was last year appointed to enquire into the nursing department. The Committee reports that they were very much impressed by the general intelligence of the nurses who came before them, and their superiority to the nurses of a few years since. Some are evidently exceptionally well fitted for the work they have undertaken; but all may be considered as affording evidence of the good judgment of Miss Brew and of her careful application of her experience when selecting probationers. Some recommendations were made, which, involving a greater outlay than the funds will at present justify, are being gradually carried out by the Board. But the Board have been gratified to learn from this impartial and most thorough enquiry, that the nurses were found contented with their surroundings, and that the system pursued at this hospital for many years past had, and still has, all the characteristics which public opinion has recently declared to be desirable in nursing institutions.

A SPECIAL PERFORMANCE.—There was a delightful performance last week at Park Hall, in aid of the Nurses' Club. Of course the usual apologies had to be made for the victims of influenza, and the epidemic kept the nurses so fully employed that many who had intended to be amongst the audience were disappointed. The Silurian Amateur Dramatic Club gave a representation of a screaming farce, "The Snowball," first. It was acted with spirit, and the fun was fast and furious. It was impossible not to join in the laughter and applause meted out to the actors. But the second part of the programme was most eagerly expected. It was called "A Dream of Sairey Gamp." The curtain rose on the Trained Nurses' Club, Buckingham Street, and a member in quiet, becoming uniform came in, and while reading *Nursing Notes* fell asleep. Had she been reading The Hospital of course she wouldn't have fallen asleep. However, it was necessary for the purposes of the dream that Morpheus should conquer, and as this pretty modern nurse lent her head on her hand, and dozed, the back of the stage withdrew, and behold, Sairey Gamp in all her disreputable glory! Betsy Prig came in to tea, and the two talked over their "sicks and monthlies," and drank their gin, and generally contrasted unfavourably with the sleeping nurse in the corner. Mrs. Nichol was to have played "Sairey," but she was unfortunately down with influenza, and the part was taken by Mr. F. Rawson Buckley, who was admirably got up, with a brilliant red tip to his nose. There were some audacious hits in this piece with regard to the present split in the nursing world, and the audience thoroughly enjoyed the jokes. Messrs. Fry and Messrs. Williamson kindly supplied some bons-bons, which were sold between the acts, and helped to contribute to what was in every way a most delightful entertainment.

A SUBURBAN ASSOCIATION.—The Camberwell District Nurses have affiliated with the Jubilee Institute, and issue a first report. "Established June 23rd, 1890, under the superintendence of Miss Ryan, assisted by Miss Spooner, and one probationer, its success—judged by the statistics found in Appendices I.-III.—has shown that a want has been met, and met in the right way." The neighbourhood knows little about district nursing, but as it sorely lacks hospital accommodation, we hope every support will be given to the new association.

SHORT ITEMS.—We are obliged to those correspondents who have sent us particulars of nurses on the register, but will not trouble them further, as the decision of the Board of Trade makes criticism superfluous.—Miss Rose Blennerhassett is on her way to Mashonaland with the Bishop of Bloomfontein, who proposes to build a hospital and put Miss Blennerhasset in charge.—Miss Wilkinson, as Miss Pratt's deputy at Derby, was presented to the Queen on the occasion of the laying of the foundation stone of the new Infirmary.—Miss Nellie Cross, daughter of the Secretary, has won the St. Bartholomew's Gold Medal.—Mr. Arthur Laing has given £100 to the Sunderland Nursing Institute.—Froken C. Goldbery, Mother-Superior of the Christiana Deaconess Home entered Kaiserwerk Institution in 1866 and has worked at Alexandria.—Each nurse will have a bedroom to herself in the new Home in connection with Derby Infirmary.

WIRRAL UNION.—A nice example of the carelessness of Guardians with regard to sick paupers is given in the accounts of the Wirral Union meetings. The Guardians at the first meeting of the newly-elected Board were visited by Mr. Murray Browne and Dr. Arthur Downes, Local Government Board Inspectors, who attended at the invitation of the Guardians, to consult with them as to the question of hospital accommodation at the workhouse. Dr. Downes remarked that Mr. Gordon Smith, architect to the Local Government Board, had pointed out the chief objections to the plans proposed by the Guardians. He himself had looked around the workhouse, and agreed with Mr. Smith. The sick were too scattered, and the nurse was too far away from them. The proposal of the Guardians was to allow about ten or twelve beds extra, but he thought they ought, according to the average, to have a detached hospital, with sixty or eighty beds in it. The Chairman explained that they had only about ninety people in the workhouse altogether. Dr. Downes replied that, under their special circumstances, perhaps forty beds would be sufficient, but to spend money on small alterations meant false economy. He did not think any of the present sick wards were suitable. They were badly ventilated, and had no baths, or water-closets, or sculleries; in fact, none of the conveniences looked for nowadays. Mr. W. Jones said their average sick had not numbered more than nineteen; and the Chairman added that the increase of sick was largely due to the Ship Canal works, and that when these were completed, in about four months' time, the pressure on the workhouse hospital would be relieved. Mr. Murray Browne said the arrangements for the sick were most primitive, and the Guardians would find it to their advantage if, instead of "messing" about, spending £100 here and £100 there, they built a satisfactory hospital. At the suggestion of Mr. Murray Browne, the books were produced for 1886—prior to the commencement of the Ship Canal works—and these showed that the average number of sick in the hospital was about thirty-two. Dr. Downes remarked that in this case the pressure on the hospital must have been great for some years, and added that, if the sick were removed from the main building, it would tend to better discipline amongst the general inmates. At this stage the Board adjourned, along with the Inspectors, to view the building, the understanding being that the matter should again be discussed at a subsequent meeting.

### Medical Petition Against Registration.

WE quote the following from the *Times* of May 22nd :—

To THE EDITOR OF THE "TIMES."

SIR,—May I ask you to be good enough to publish in your next impression the accompanying petition, which has been presented to the President of the Board of Trade. I would desire to draw your attention to the fact that the signatories to this petition are for the most part medical men who are or have been directly engaged in teaching and training nurses, and have therefore far greater right to speak with authority upon the matter at issue than those whose names appeared to the letter you published on April 30 in support of the action of the Royal British Nurses' Association, who, though some of them are distinguished men in their profession, cannot be said to have identified themselves with nurse-training. Also that the register lately published by the association itself confirms in the most convincing manner the objections which have been urged against it, and illustrates very forcibly the evils which must result from the legal recognition of such a register.

I am, Sir, yours obediently,
J. G. WAINWRIGHT, Treasurer of
St. Thomas's Hospital.

St. Thomas's Hospital, May 21.

To the Right Hon. the President of the Board of Trade.

The humble petition of the undersigned physicians and surgeons, who are directly concerned in, or have given large attention to, the education of nurses in the nurse-training schools of Great Britain,

Showeth as follows :—

It has recently come to our knowledge that a self-constituted body of ladies and gentlemen, styling itself the British Nurses' Association has applied to the Board of Trade for a licence under the 23d section of the Companies Act of 1867 and that under Clause 8 of its memorandum of association it proposes—

"To form, control, and carry on

"(1) A register of trained nurses.

"(2) A register of certificated midwives, and to determine from time to time what tests shall be specified by candidates for registration, as evidence that they possess skill and knowledge in their profession;" or, in other words, that the British Nurses' Association aims at exercising, under cover of a licence from the Board of Trade, the power of granting certificates of competency in nursing, and so of controlling the education of nurses, a power such as has hitherto only been conferred on really representative bodies by Royal Charter or by special Act of Parliament, and is not within the scope of the Companies Acts.

Against this scheme of registration we desire to protest on the following grounds :

(1) That a self-appointed association, such as the British Nurses' Association, is not a fitting or competent authority to determine, in the interest either of the nursing profession or of the general public, who should be put on the register, or who should be excluded from it.

(2) That no written or oral examination of nurses in the technical details of their duties can possibly lead to any approximate estimate of their real fitness and competence as nurses, and least of all an examination conducted apart from hospitals, and by persons not specially qualified, for a nurse's qualifications depend mainly on practical experience, or natural gifts and moral qualities, which a mere examination, however well conducted, can never adequately test.

(3) That the effect of the proposed register of nurses, by granting certificates of competency professing to be authoritative, while being necessarily imperfect and untrustworthy, would be to mislead instead of guiding both the public and medical practitioners and to lower the standard of nursing by placing numbers of insufficiently trained and inferior nurses on the same level as their highly-trained and thoroughly competent sisters.

(4) That the authorities of the nurse-training schools are alone in a position, from their experience and special knowledge, and from their intimate acquaintance with the individual nurses who have been trained under their care, to certify who are fit and properly-trained nurses, and that the certificates of efficiency given by them are sufficient, and are infinitely more valuable and trustworthy than any certificates otherwise acquired could possibly be.

(5) That no association having for its object to test and guarantee by certificate the educational and other qualifications of its members has ever yet had accorded to it such powers and privileges as the British Nurses' Association aims at acquiring, until it has been shown by actual results that its action has been beneficial to the public and to the body it purports to represent, and that it has the support of the leading members, as well as of the large majority of the rank and file, of that body. The large nursing-training school of Great Britain, including the Nightingale School, to whose labours the vast improvement which has of late years taken place in the education and status of nurses is wholly attributable, are, almost without exception, unrepresented in the British Nurses' Association and are opposed to its registration scheme.

SIR W. BOWMAN, F.R.S., Member of Council of Nightingale Fund; J. S. BRISTOWE, M.D., F.R.S., Senior Physician to St. Thomas's Hospital and Lecturer to the Nightingale Training School; JOHN CROFT, F.R.C.S., Senior Surgeon to St. Thomas's Hospital, Lecturer on Anatomy and Surgery to the Nightingale Training School; SYDNEY JONES, M.B., Consulting Surgeon to St. Thomas's Hospital and member of the Council of the Royal College of Surgeons; H. GERVIS, M.D., Consulting Obstetric Physician to St. Thomas's Hospital; THOMAS BRYANT, President of the Royal College of Surgeons, England, Consulting Surgeon to Guy's Hospital; G. NEWTON PITT, M.D., Physician to Guy's Hospital, formerly Instructor of Nurses; J. W. WASHBOURNE, M.D., Physician to Guy's Hospital and Instructor of Nurses; E. C. PERRY, M.D., ditto, formerly Instructer of Nurses; W. ARBUTHNOT LANE, F.R.C.S., Assistant Surgeon ditto, Instructor of Nurses; J. C. STOELE, M.D., Medical Superintendent Guy's Hospital; W. H. ALLCHIN, M.B., Physician to Westminster Hospital and to Westminster Training School and Home for Nurses; THOMAS BOND, F.R.C.S., Surgeon to Westminster Hospital; F. DE HAVILLAND HALL, M.D., Physician to Out-patients, Westminster Hospital; GEORGE COWELL, F.R.C.S., Senior Surgeon ditto; J. B. POTTER, M.D., Obstetric physician, ditto; C. N. MACNAMARA, Surgeon to Westminster Hospital, and member of Council of Royal College of Surgeons; STANLEY BOYD, M.B., F.R.C.S., Surgeon to Charing Cross Hospital and Lecturer to Nurses; FREDK. WILLCOCK, M.D., Physician to Out-patients, Charing Cross Hospital, and Lecturer to Nurses; JOHN CURNOW, M.D., Dean of the Medical Faculty, King's College, and Lecturer to Nursing Staff; HENRY G. SUTTON, M.D., Physician to the London Hospital; STEPHEN MACKENZIE, M.D., ditto; FREDK. TREVES, F.R.C.S., Surgeon to the London Hospital, late Lecturer to the Nursing School; A. ERNEST SANSOM, M.D., Physician ditto; JAMES ANDERSON, M.D., Assistant Physician ditto, and Lecturer to the Nursing School; CHARLES MANSELL MOULLIN, F.R.C.S., Surgeon to the London Hospital; T. GILBART SMITH, M.D., Physician ditto; WARREN TAY, F.R.C.S., Senior Surgeon ditto; FREDK. S. EVE, F.R.C.S., Assistant Surgeon ditto; THOMAS H. OPENSHAW, F.R.C.S., ditto; FREDK. H. SMITH, B.A., M.B., Medical Registrar, London Hospital; W. A. MACKINNON, C.B., Director-General Army Medical Staff; J. B. C. READE, C.B., Surgeon-General ditto; W. NASH, M.D., Brigade Surgeon Medical Staff; S. B. PARTRIDGE, Deputy Surgeon-General ditto; CHARLES D. MADDEN, Surgeon-General ditto; SIR THOMAS LONGMAN, C.B., Surgeon-General (retired), Professor of Military Surgery, Army Medical School; SIR W. AITKEN, M.D., F.R.S., Professor of Physiology Army Medical School; J. LANE NOTTER, M.A., M.D., Professor of Military Hygiene ditto; A. M. DAVIES, M.R.C.S., Assistant Professor of Military Hygiene ditto; W. D. STEVENSON, Assistant Professor of Military Medicine ditto; JOHN E. MORGAN, M.D., M.A., Professor of Medicine in Victoria University, Manchester; JAMES ROSS, M.D., LL.D., Physician to Manchester Royal Infirmary, Professor of

Medicine Victoria University; THOMAS HARRIS, M.D., Assistant Physician Manchester Royal Infirmary; JUDSON S. BURY, M.D., Assistant Physician ditto; D. J. LEECH, M.D., Physician ditto; JAMES BARR, M.D., Physician, Northern Hospital, Liverpool; EDWARD H. DICKINSON, M.D., Senior Physician ditto; J. DRESCHFELD, M.D., Physician Manchester Royal Infirmary, Professor of Pathology, Owens College; T. CUMING ATKIN, M.D, House Physician Northern Hospital, Liverpool; CHAUNCY PUZEY, M.R.C.S., Surgeon ditto; DANIEL HARRISON, F.R.C.S., Surgeon ditto; G. G. HAMILTON, M.B., Surgeon ditto: R. D. MOTHERSOLE, M.B., House Surgeon, ditto; W. ALEXANDER, F.R.C.S., Chairman of Medical Board Royal Southern Hospital, Liverpool; JOHN CAMERON, M.D., Senior Physician ditto; HORACE SWETE, M.D., Physician Worcester General Infirmary; GEO. W. CROWE, M.D., Physician ditto; E. WALPOLE.SIMMONS, M.D., Physician ditto; H. G. BUDD, Surgeon ditto; T. BATE, Surgeon ditto; G. HYDE, Surgeon ditto; G. R. J. FLETCHER, House Surgeon ditto; THOMAS ANNANDALE, Surgeon Edinburgh Royal Infirmary, and Professor of Clinical Medicine, University of Edinburgh; JOHN CHIENE, Surgeon to the Edinburgh Royal Infirmary and Professor of Systematic Surgery, University of Edinburgh; JOHN DUNCAN, President of the Royal College of Surgeons, Edinburgh, and Surgeon to the Royal Infirmary; A. G. MILLER, Surgeon to the Royal Infirmary, Edinburgh; CHARLES W. CATHCART, Assistant Surgeon ditto; JAMES HODSDON, Assistant Surgeon ditto; JAMES FINLAYSON, Physician to Glasgow Western Infirmary, and formerly Lecturer to Nurses; WM. J. FLEMING, Surgeon Royal Infirmary, Glasgow, and late Lecturer on Surgical Nursing, Royal and Western Infirmary.

### Everybody's Opinion.

*[Correspondence on all subjects is invited, but we cannot in any way be responsible for the opinions expressed by our correspondents. No communications can be entertained if the name and address of the correspondent is not given, or unless one side of the paper only be written on.]*

### THE NEED FOR PENSION FUNDS.

"MISS S. J." writes:—Seeing what "A District Superintendent" writes, I wish to draw attention to, and say a word for a sadly neglected class, namely, the ladies who have arrived at old age, having spent the best years of their lives as governesses, and are totally without any means of living. With incomes, out of which it would have been impossible for them to save anything, required to dress well and provide herself with board and lodging during the holidays often amounting to three months in the year. Why does no one come forward to lend a helping hand to start a fund for pensions for them? The enormous sums that are collected for "girls' clubs," as well as boys' clubs! The places that are built for the amusement of the working classes, while thousands of poor ladies are left to utter destitution, no shelter for them but the workhouse, brought up in refinement, and left without any means or shelter whatsoever! Will not some lady come forward for the benefit of this cruelly neglected class, for the most part daughters of professional gentlemen, who, from no fault of their own, are utterly destitute?

### PENSIONS FOR SERVANTS.

"VERITAS" writes: In a late issue a correspondent proposed that a pension fund be raised for domestic servants, stating that such would benefit the employers as well as the domestic, viz., by better work, affection, and longer service on the part of the employed. As matters now stand, I think that domestic servants as a rule (there are, of course, exceptions) are almost the most despised class of workers at present existing. Why not, before starting a pension fund, urge the employers to place a little more trust and confidence in their servants? I am sure the employers would not be losers by it, in fact it would help to raise a class of servants (there are plenty now existing) who would serve from the highest motive, "not as eye servants, but in singleness of heart fearing God."

### ST. JOHN'S CONVALESCENT HOME.

MISS BORRADAILE writes: My attention has been drawn to a paragraph in THE HOSPITAL of April 25th concerning the St. John's Home for Convalescent and Crippled Children, Brighton, which states that the receipts for the past year have exceeded the expenditure by £254 16s. 6d. The balance-sheet certainly shows a balance of that amount to the credit of the Home at the close of 1891, but a large sum was required to meet a call from the builders at the commencement of the present year, so had to be kept in hand, otherwise it would have at once gone to meet the large debt of nearly £2,000 with which the Home is still struggling. It will thus be seen that the finances are not quite in the flourishing condition which a first glance of the balance-sheet might convey. A substantial and commodious house has been built to accommodate between sixty and seventy children, and girls training for service, at the cost of over £5,000, and the Home is still greatly in need of the earnest assistance of all its friends to meet its heavy obligations and to place it on a more permanent basis.

### DISTRICT NURSES AT HARTLEPOOL.

DR. A. E. MORISON writes: I notice in THE HOSPITAL of the 16th you state that Nurse Mary Mills is the first District Nurse in Hartlepool. There is a slight error in this. She is the first in West Hartlepool, but Hartlepool claims priority to having a District Nurse, Nurse Margaret Schwappe being at work quite three months before the movement originated in West Hartlepool. Besides Nurse Schwappe there is under her care and supervision a Nursing Guild, composed of ladies who devote their spare time to nursing the poor in their own homes. To show how thoroughly and efficiently the work is carried on I enclose you our first report.

### PENNIES A NECESSITY.

"A SPINSTER" writes: May I call your attention to what may be a serious inconvenience to invalids or delicate girls travelling specially by express trains? Alighting at a first-class station in the Midland counties a few days ago, I entered the first-class ladies' waiting-room, and found I could not avail myself of the usual conveniences until I had put a penny in the slot. I had no penny; any small silver coin, such as I should have given an attendant, was useless. There was no attendant there to give change. The train I was travelling by would not stop for two hours, and then at an inconvenient station for a very few minutes. Whether this system prevails in the second and third-class waiting-rooms I do not know, but I hope you will kindly use your influence to expose so undesirable an economy on the part of the railroads, and, at all events, to warn women what they may expect, as all may not be so outspoken as a spinster of fifty.

### Warwick Association.

THE annual meeting of this Association was held last week with Major Fosbery in the chair. Mrs. Campbell read the report. The Committee engaged, on May 5, 1890, the services of a trained hospital nurse, who has performed her duties in a very efficient manner, and Nurse Gazey's skill and kindness have been much appreciated by the poor and by the medical practitioners whose patients she has attended. The nurse's duties comprise dressing wounds, applying bandages or poultices, taking temperature, making the patient's bed, and seeing that the doctor's orders are carried out, proper nourishment and stimulants supplied, and necessary linen lent. In some urgent cases she visits twice or three times daily, in others, not serious but requiring occasional attention, she goes once a day or less frequently. The question of the need for a second nurse was debated.

Mrs. Campbell explained that the number of cases attended in the four parishes were: St. Mary's, 52; St. Paul's, 44; St. Nicholas' 15; and Emscote, 6. These figures showed that if there had been more illness in two of the parishes, as in the other two, it would have been almost impossible for the nurse to have carried out her work. If sufficient funds are forthcoming another nurse will be engaged before next winter.

### The Hospitals Association.

The Marquis of Carmarthen, M.P., has consented to take the chair at the annual meeting of the Hospitals Association to be held on Tuesday, June 9, at 3 p.m., at 140, Strand. This is good news, and a good report may be expected, for most of our readers know what interesting meetings this Association has held during the winter season, and many must have seen the ambulances it has stationed, or is stationing all over London. The Association has always been very friendly to nurses and numbers amongst its members the Matrons of nearly all the large London hospitals and infirmaries.

### Appointments.

Pendlebury Hospital.—Miss Measures has been appointed Night Superintendent at the Children's Hospital, Pendlebury. She is a Guy's certificated nurse, and has lately been in charge of a ward at the Kent and Canterbury Hospital, from which place she received excellent testimonials.

St. George's in the East Infirmary.—Miss E. A. Wickham has been appointed Night Superintendent of the above infirmary. Miss Wickham holds the Guy's three years' certificate, and the diploma of the L.O.S.

### Presentation.

Miss A. Ross having lately been elected Matron of the Royal Infirmary, Newcastle-on-Tyne, she was, on May 19th, presented with a handsome marble timepiece from the nursing staff of the hospital. Miss Ross entered the institution first as a probationer seven years ago, and since then has filled successively the posts of Head Nurse and Night Superintendent. It is as a mark of their high regard and esteem for Miss Ross that her fellow-nurses have presented her with the timepiece, and as a means of conveying to her their most hearty congratulations on her appointment to the Matronship after so many years of work with them in the wards.

### Death in Our Ranks.

Many of our readers will hear with deep regret of the sudden death of Miss Florence Emily Derham, at the early age of 33. Miss Derham was a trained nurse of wide experience, her chief work being at Charing Cross Hospital, and at Bristol and Leamington. Some three years ago Miss Derham and her friend, Miss Dunn, started a Convalescent Hospital for Children at Bushey Heath, which has been most useful and most successful. On May 19th Miss Derham attended a reception at a friend's house, and while there died almost instantaneously from failure of the heart's action. While sympathising deeply with all Miss Derham's friends, we hope their friendship will take the practical form of carrying on the good work she had so nobly begun.

### MESSRS. SOUTHALL BROS.' SPECIALITIES.

We have often recommended to nurses and to all women many of the articles patented by this firm, but we want specially to address a word to private and monthly nurses about the knapkenettes for infants and the sanitary sheets. Southall's sanitary sheets are to be highly recommended for use in illness, as they are of downy softness and great elasticity, comfortable, cleanly, agreeable, and thoroughly absorbent. In cases of sickness, where the patient cannot be moved, these sheets are invaluable for protecting the bedding. They are also of the greatest service for use in infants' cots, to supersede the mackintosh. The prices of these are 6d., 1s., and 2s. each. All these goods may be obtained at most chemists and underlinen shops, but if there is any difficulty, immediate attention may be assured by writing direct to the Lady Manager, 17, Bull Street, Birmingham.

### Wants and Workers.

[Under this heading, we propose to try for a few weeks whether we can be useful to our readers in making the wants of some known to others who are willing to do what work they can to aid the great cause of curing and cheering the sick.]

### Wants.

*A Piano.*—Miss Janie Charles, Hon. Secretary to the Santa Claus Society, Stormont, Hampstead Lane, Highgate, appeals for a piano or harmonium for the new Convalescent Hospital for Children.

*A. G. T.*—The Nurses' Co-operation, 8, New Cavendish Street, Portland Place, London, W., will be very grateful for the two weekly papers you mention.

### Workers.

*Papers.*—I have piles of illustrated and comic papers I would send to any fever hospital which wants them.—*A. G. T.*

*Ladies' Belt.*—I have an abdominal belt I would send to any district nursing society needing such an article for one of its patients.—*V. H.*

*A Gift.*—Would any nurse or nursing institution like to have The Hospital from March, 1888, up to end of last volume? They are in weekly parts, and in good condition.—*A. G.*

### Notes and Queries.

To Correspondents.—1. Questions or answers may be written on post-cards. 2. Advertisements in disguise are inadmissible. 3. In answering a query please quote the number. 4. A private answer can only be sent in urgent cases, and then a stamped addressed envelope must be enclosed. 5. Every communication must be accompanied by the writer's full name and address, not necessarily for publication. 6. Correspondents are requested to help their fellow nurses by answering such queries as they can.

### Queries.

(13) *California.*—Can any nurse give me information about nursing work, especially monthly nursing, in California? Also about the climate, fees, and the best part to go to?—*Private Nurse.*

(14) *Home for Consumptive.*—Can any reader tell me of a permanent home where a consumptive girl could be taken free, or for small payment?—*M. A. B. Y. S.*

### Answers.

*Nurse Elizabeth.*—The Jubilee Institute would not give you the general training you require; but you could get a year at Chichester Infirmary for £10, or a year at Whitechapel Infirmary, neither paying nor being paid. If neither of these have vacancies, try Royal Hull Infirmary, fee 15 guineas for the year.

*Emmeline.*—Eighteen is the very youngest at which a nurse is taken into a children's hospital, and you had far better wait until you are twenty.

*M. M.*—You will have the advantage of the badge and of the name of being a Queen's nurse. There is no compulsion whatever about joining the Pension Fund.

*Dove.*—"A Guide to District Nursing," by Mrs. Dacre Craven. (Macmillan. Price 2s. 6d.)

*Miss H.*—The editor of the *Nightingale* answered your letter in the number for April 11th.

*Physician.*—You will find exactly what you require in a small book (price about a shilling), "Training Schools for Nurses," by W. G. Thompson, M.D., published by Putnams. It has an introductory chapter dealing with the past. If you have any difficulty in procuring the book, we shall be happy to lend you our copy. "The Life of Agnes Jones" would also be useful to you. The Hospital for October 1st and 29th, 1887, had articles on "Sick Nursing in the Sixteenth Century."

### Amusements and Relaxation.

**SPECIAL NOTICE TO CORRESPONDENTS.**

**Second Quarterly Word Competition commenced April 4th, ends June 27th, 1891.**

Competitors can enter for *all* quarterly competitions, but no competitor can take more than *one first prize* or *two* prizes of any kind during the year.

N.B.—Word dissections must be sent in WEEKLY not later than the first post on Thursday to the *Prize Editor*, 140, Strand, W.C., arranged alphabetically, with correct total affixed.

The words for dissection for this, the NINTH week of the quarter, being

"EPIDEMIC."

| Names. | May 21st. | Totals. | Names. | May 21st. | Totals. |
|---|---|---|---|---|---|
| Christie | 114 | 330 | Ivanhoe | 91 | 278 |
| Patience | — | 214 | Weta | — | — |
| Agamemnon | 115 | 333 | Lady Betty | 94 | 298 |
| Hope | 112 | 332 | Mortal | — | 76 |
| Reldas | 120 | 334 | Little Eliza | — | — |
| Lightowlers | 98 | 306 | Dove | — | 95 |
| Nurse J. S. | 99 | 276 | Ladybird | — | — |
| Qu'appelle | — | — | Psyche | 107 | 292 |
| Jenny Wren | 58 | 260 | Ugug | 72 | 229 |
| Wyameris | 120 | 335 | Harrie | 56 | 109 |
| Paignton | 89 | 275 | Grannie | 101 | 271 |
| Theta | — | 206 | Eale | — | 169 |
| Success | — | 17 | Grimalkin | — | 53 |
| Tired | — | 136 | Nurse G. P. | 43 | 68 |
| M. G. | — | 188 | | | |

### Notice to Correspondents.

N.B.—All letters referring to this page which do not arrive at 140, **Strand, London, W.C.**, by the *first post on Thursdays*, and are not addressed PRIZE EDITOR, will in future be disqualified and disregarded.

